# Exploring Causal Network Complexity in Industrial Linkages: A Comparative Study

**DOI:** 10.3390/e27020209

**Published:** 2025-02-17

**Authors:** Yongmei Ding, Chao Huang, Xubo Feng

**Affiliations:** Department of Mathematics and Statistics, College of Science, Wuhan University of Science and Technology, Wuhan 430065, China; zznwwyhc@gmail.com (C.H.); ball1133@foxmail.com (X.F.)

**Keywords:** industry linkage, PCM, causal networks, network resilience

## Abstract

Industrial linkages play a crucial role in sustaining industrial agglomerations, driving economic growth, and shaping the spatial architecture of economic systems. This study explores the complexity of causal networks within the industrial ecosystems of China and the United States, using high-frequency economic data to compare the interdependencies and causal structures across key sectors. By employing the partial cross mapping (PCM) technique, we capture the dynamic interactions and intricate linkages among industries, providing a detailed analysis of inter-industry causality. Utilizing data from 32 Chinese industries and 11 United States industries spanning 2015 to 2023, our findings reveal that the United States, as a global leader in technology and finance, exhibits a diversified and service-oriented industrial structure, where financial and technology sectors are pivotal to economic propagation. In contrast, China’s industrial network shows higher centrality in heavy industries and manufacturing sectors, underscoring its focus on industrial output and export-led growth. A comparative analysis of the network topology and resilience highlights that China’s industrial structure enhances network stability and interconnectivity, fostering robust inter-industry linkages, whereas the limited nodal points in the United States network constrain its resilience. These insights into causal network complexity offer a comprehensive perspective on the structural dynamics and resilience of the economic systems in both countries.

## 1. Introduction

In the contemporary global economic landscape, the United States and China dominate in a “two-pillar” structure. In 2023, the United States remained the world’s largest economy with a GDP of USD 21.8 trillion, accounting for about 24.5% of the global GDP, while this was followed by China’s 15.5%. As two of the world’s largest economies with significant interconnections, cooperation and competition are inevitably engaged. Concurrently, the world is undergoing unprecedented changes, where global dynamics are increasingly interrelated and interactive. Events such as the onset of the United States–China trade war in 2018, the outbreak of the COVID-19 pandemic in late 2019, the Russo-Ukrainian conflict in early 2022, and the tensions in the Middle East in 2023 have all had significant repercussions for them. Against the backdrop of economic globalization and the concept of a global community with a shared destiny, countries and sectors are becoming increasingly interconnected.

Industries’ interconnectedness significantly influences the international competitiveness of a country or region. Highly interconnected industries often present more opportunities for collaboration and innovation. Enhanced connectivity between sectors facilitates the transfer of knowledge and technology, fostering overall economic growth [1,2]. This interconnectedness is further amplified by the advent of digital technologies and the development of digital and spatial affordances, which contribute to the formation of complex industrial and entrepreneurial ecosystems [3]. However, such high interconnectivity also renders industries more susceptible to unexpected events, such as disruptions in global supply chains, which can propagate damages across multiple sectors and consequently exert a greater impact on the overall economy [4]. Incorporating causality-based metrics significantly enhances our understanding of the fault propagation, root cause tracing, and network dynamics in industrial systems. Furthermore, adaptive techniques such as CoCoLasso provide robust tools for handling high-dimensional data and measurement errors, as highlighted in previous research [5,6,7,8]. Industries’ interconnectedness offers a systematic approach to capturing, quantifying, and interpreting the causal interactions, with potential applications in fault diagnosis, system optimization, and policy design.

Governments manipulate the interlinkages between industries through industrial policy and interventions, which may either reinforce or diminish these linkages to achieve particular economic and social objectives [9]. In this context, the role of strategic management within these interconnected ecosystems becomes paramount. As industries increasingly adopt new business models and digital enterprises, the structure of industrial linkages is reshaped, influencing the value capture and organizational dynamics within these networks [10]. In the stage of globalization, highly interconnected economies often enjoy enhanced competitiveness by leveraging their access to global supply chains and markets [11], which drive industrial upgrading and indirectly stimulate the exploration and application of data-driven correlations [12,13,14].

Innovative data-driven fault detection methodologies, such as Principal Component Analysis (PCA), Partial Least Squares (PLS), and Canonical Correlation Analysis (CCA), have been extensively researched and developed, showcasing their potential in various applications [15,16,17]. The increasing complexity of industrial networks and the critical role of technology in shaping their interdependencies have been highlighted, underscoring the importance of understanding the dynamic nature of these networks [18,19]. Studies have also explored how industry linkages contribute to innovation and competitiveness, emphasizing the role of industrial ecosystems in fostering these dynamics [20,21]. Causal metrics, when incorporated into industrial network models, can capture the directionality and strength of relationships, which are critical for diagnosing fault propagation paths [22,23].

Statistical inference methods, such as Granger Causality (GC), Vector Autoregression (VAR), Transfer Entropy (TE), Structural Equation Modeling (SEM), and Bayesian Networks (BNs), perform feature extraction and hypothesis tests to judge the causal relationships for time series or probability structure data [24,25,26,27]. Another important observation is the role of causality in optimizing network robustness. The inclusion of causality structures into time-varying network analyses, as suggested by Carlos-Sandberg and Clack [28], offers a powerful mechanism for monitoring system performance and mitigating risk. Moreover, the synergy between causality-based approaches and neural network models further expands their application potential, particularly in soft sensing and fault prediction [29]. However, these methods primarily focus on identifying patterns, correlations, and latent structures and are not inherently designed to distinguish between direct and indirect causality [30]. This limitation can lead to misjudging true direct causal relationships, especially within the nonlinear and dynamic industrial systems that characterize modern economies.

In this paper, we have employed partial cross mapping (PCM) [31] to differentiate direct causality within industrial systems and measure the inter-industry linkages through phase space reconstruction. We have identified key nodes in the industrial structures and compared the linkages between China and the United States via a causal network. Industrial systems with numerous interdependent nodes have been effectively modeled using linkage indicators, incorporating both direct and indirect causal relationships, elucidating the asymmetry of causality, and enhancing the accuracy of the industrial interrelations. Social Network Analysis (SNA) has been employed to examine the intricate characteristics of industry nodes to provide a more comprehensive depiction of inter-industry causal linkages. The robustness of industrial networks to deliberate and random attacks has also been further measured.

## 2. Methodology

### 2.1. PCM (Partial Cross Mapping)

An articulated integration of three tools from nonlinear dynamics and statistics—phase space reconstruction, mutual cross mapping, and partial correlation—named partial cross mapping is an effective way to differentiate direct causations from indirect ones when the variables of the underlying dynamical system are non-separable and interact weakly or moderately. Take three time series $X={\left\{x_{t}\right\}}_{t=1}^{L}$, $Y={\left\{y_{t}\right\}}_{t=1}^{L}$, and $Z={\left\{z_{t}\right\}}_{t=1}^{L}$ with the length *L*; three shadow manifolds can be gained using Takens–Mane’s delay-coordinate embedding technique, as $\left(M_{X},M_{Y},M_{Z}\right)$—$M_{X}={\left\{x_{i}\right\}}_{i=r}^{L},M_{Y}={\left\{y_{i}\right\}}_{i=r}^{L},M_{Z}={\left\{z_{i}\right\}}_{i=r}^{L}$—where the state vectors are given as follows:

(1) $$\begin{array}{cc} x_{i} & =(u_{i},u_{i+\tau_{x}},u_{i+2\tau_{x}},\ldots ,u_{i+\left(m_{x}-1\right)\tau_{x}})\\ y_{i} & =(v_{i},v_{i+\tau_{y}},v_{i+2\tau_{y}},\ldots ,v_{i+\left(m_{y}-1\right)\tau_{y}})\\ z_{i} & =(w_{i},w_{i+\tau_{z}},w_{i+2\tau_{z}},\ldots ,w_{i+\left(m_{z}-1\right)\tau_{z}}) \end{array}$$

where $m_{x},m_{y}$, and $m_{z}$ are the embedding dimensions, and $\tau_{x},\tau_{y}$, and $\tau_{z}$ are time lags, which are deduced using False Nearest Neighbor (FNN) and Delayed Mutual Information (DMI), respectively. Here, we set $\tau_{x},\tau_{y}$ and $\tau_{z}$; $m_{x},m_{y}$, and $m_{z}$; and $r=max_{\xi }\left\{1+\left(m_{\xi }-1\right)\tau_{\xi }\right\}$, with ξ=(x,y,z).

Determine a fixed number of nearest neighbors for state $x_{i}$ (usually set to *m* + 1). For $u_{t}$, find its nearest neighbors in $M_{X}$, denoted as $N_{X}\left\{u_{t}\right\}$.Then, map to $M_{Y}$, where the corresponding nearest neighbor set in $M_{Y}$ is $N_{Y|X}\left\{v_{t}\right\}$, and $N_{Y|X}\left(v_{t}\right)$ = $\left\{v_{t^{\prime }}|u_{t^{\prime }}\in N_{X}\left(u_{t}\right)\right\}$. Finally, calculate the weighted average of $N_{Y|X}\left\{v_{t}\right\}$ to obtain the mapping estimate ${\hat{y_{i}}}^{x_{i}}$. This process can be used to obtain the estimated time series ${\hat{Y}}^{X}$ for *Y*.

Similarly, $N_{Z|X}\left(w_{t}\right)$ and ${\hat{z}}_{i}^{x_{i}}$ can be obtained. Next, map the nearest neighbors in ${\hat{z}}_{i}^{x_{i}}$ directly to $M_{Y}$, resulting in the nearest neighbor set $N_{Y|X,Z}\left(v_{t}^{\prime }\right)$, from which the mapping estimate ${\hat{y}}_{i}^{{\hat{z}}_{i}^{x_{i}}}$ can be obtained. This process can be used to obtain the estimated time series ${\hat{Y}}^{{\hat{Z}}^{X}}$ for *Y*.

The detailed form of the partial correlation coefficient between *Y* and *X* conditioned with *Z* is given as follows:

(2) $$Pcc\left(Y,{\hat{Y}}^{X}|{\hat{Y}}^{{\hat{Z}}^{X}}\right)=\frac{Corr\left(Y,{\hat{Y}}^{X}\right)-Corr\left(Y,{\hat{Y}}^{{\hat{Z}}^{X}}\right)Corr\left({\hat{Y}}^{X},{\hat{Y}}^{{\hat{Z}}^{X}}\right)}{\sqrt{\left(1-Corr{\left(Y,{\hat{Y}}^{{\hat{Z}}^{X}}\right)}^{2}\right)\left(1-Corr{\left({\hat{Y}}^{X},{\hat{Y}}^{{\hat{Z}}^{X}}\right)}^{2}\right)}}$$

where Corr(*) represents the correlation between the variables.

The correlation coefficient in the mutual cross mapping structure is measured as follows:

(3) $$R_{c}=\left|Corr\left(v_{t},{\hat{v_{t}}}^{u}\right)\right|$$

where ${\hat{v_{t}}}^{u}=E\left({\hat{N}}^{u}\left(v_{t}\right)\right)$, ${\hat{v_{t}}}^{u}$ is the mapping from $u_{t}$, and E(·) is the appropriately weighted average at all points in each set. Given a threshold *T*, according to the rule of mutual correlation, a causal influence from *X* to *Y* exists if $R_{C}$ is larger than *T*.

In real-world systems, causal relationships between variables frequently involve time lags. Therefore, when we evaluate the causal link between two variables, it is essential to determine the optimal time lag to maximize the causal coefficient. To effectively address the challenge of causation transitivity, we introduce the direct causal inference index, defined as follows:

(4) $$R_{D}=\left|Pcc\left(y_{t},{\hat{y_{t}}}^{x}|{{\hat{y}}_{t}}^{{\hat{Z}}^{X}}\right)\right|$$

where ${{\hat{y}}_{t}}^{{\hat{Z}}^{X}}$ is extracted as the indirect causality through *Z*, and Pcc(·) is the partial correlation coefficient between the first two variables.

According to the statistical principles, $R_{C}\geq R_{D}$ always applies. Suppose a empirical threshold *T* is given; the causal relationship between *X* and *Y* can be deduced via the following frame:

(5) $$\left\{\begin{array}{c} direct\;causality\;of\;Y\to X\;\;\;\;\;\;\;\;if\;R_{C}\geq R_{D}\geq T\\ indirect\;causality\;of\;Y\to X\;\;\;\;\;\;\;if\;R_{C}\geq T\geq R_{D}\\ absent\;causality\;of\;Y\;\to X\;\;\;\;\;\;\;if\;T\geq R_{C}\geq R_{D} \end{array}\right.$$

### 2.2. Linkage Indicators

‘Linkage indicators’ measure the strength of the causal relationships among industries as derived from the partial cross mapping (PCM) technique. This differs from the traditional interpretation in Input–Output Analysis (IOA), where linkage effects typically refer to indirect ripple effects in the forward or backward directions, quantified using inter-industry transaction matrices. Our indicator captures both the direct and indirect causal linkages within a network framework, emphasizing the dynamics of inter-industry influence rather than economic transactions alone.

(A) **Degree Centrality.** Degree centrality measures the number of connections of a node in the network, named the degree of a node, which is its connection with other nodes. Higher degree centrality plays a pivotal role and is calculated as expressed in (6).
  (6) $$C_{D}\left(v\right)=\frac{deg(v)}{N-1}$$
  where $C_{D}\left(v\right)$ is the degree centrality of the node, deg(v) is the degree of the node, and *N* is the total number of nodes in the network.
(B) **Betweenness Centrality.** Betweenness centrality measures the role of nodes as intermediaries on the shortest path in the network. Nodes with high intermediate centrality are usually located on the information flow path and control the information propagation and connect important nodes in different parts. The formula is shown in (7).
  (7) $$C_{B}\left(v\right)=\sum_{s\neq v\neq t}\frac{\sigma_{st}\left(v\right)}{\sigma_{st}}$$
  where $\sigma_{st}$ is the total number of shortest paths from node *s* to *t*, while $\sigma_{st}\left(v\right)$ is the number of those paths passing through node *v*.
(C) **Closeness Centrality.** Closeness centrality measures the average distance between nodes and other nodes. Nodes with high compact centrality are closer to other nodes in the network, and it is measured using (8).
  (8) $$C_{C}\left(v\right)=\frac{N-1}{\sum_{u\neq v}d\left(v,u\right)}$$
  where $C_{C}\left(v\right)$ is the closeness centrality of node *v*, and *d*(*v*,*u*) is the shortest path length between nodes *v* and *u*.

### 2.3. Resilience Measurement

In evaluating the robustness of industrial networks, we selected two characteristics related to random and deliberate attacks on network nodes to assess their impact on network efficiency.

In a random attack, the attacker selects a node or edge in the network to attack, while the selected target is random and there is no specific strategy. The main purpose of random attacks is to test the resilience and robustness of the network to understand how the network behaves when it is subjected to random damage, which helps us understand the sensitivity of the network structure under the condition of random attacks.

In a deliberate attack, the attacker selects a specific node or edge to attack, usually based on the network topology or the criticality of the network function. The goal of a deliberate attack may be to disrupt specific parts of the network, paralyze critical nodes, disrupt network communications, or impede the transmission of information. The attacker exploits a vulnerability or structural weakness in the network to carry out an attack in order to achieve their objectives.

Here, we employ two indices, named the global network efficiency and maximum connected subgraph size, to capture the feature of the resilience of industrial linkages.

(A) **Global network connectivity.** This describes the overall communication efficiency based on the shortest path length between nodes in the network and reflects the speed of information transfer in the network, with the equation for its calculation given in (9).
  (9) $$E=\frac{1}{N(N-1)}\sum_{i\neq j}\frac{1}{d_{ij}}$$
  where *N* is the number of nodes in the network and $d_{ij}$ is the shortest path length between node *i* and *j*. The value ranges from 0 to 1. This index tends towards 0 if the network edges are sparse, while it tends towards 1 if the network is highly connected.
(B) **Maximum connectivity subgraph scale.** This index is defined as the ratio of the number of nodes contained in the maximum connected subgraph to the total number of nodes in the network, noted as *S*. As the network is constantly attacked and gradually fragmented, *S* can further reflect the stability shown by the network collapse process. The measurement of *S* is shown in (10).
  (10) $$S=\frac{L}{N(1-f)}$$

where *f* indicates the proportion of nodes removed after the attack, and *f* is $f_{0}$ the critical value for network collapse. *L* is the number of nodes contained in the maximum connected subgraph.

## 3. Empirical Analysis

### 3.1. About the Data

The bilateral relationship between China and the United States stands as one of the most critical in today’s global landscape. In the United States, industry classifications are commonly aligned with the standards set by S&P Dow Jones Indices, categorizing the primary industries into 11 sectors, as shown in Table 1. The data utilized in this analysis are sourced from the official S&P Dow Jones Indices website, accessible at S&P Global.

The industry classification typically follows the Shenwan Industry Classification in China, developed by the Research Institute of Shenwan Hongyuan (http://www.swsindex.com/). Prior to 2020, the Shenwan classification system identified 28 primary industry categories. In 2021, the number of primary industries was adjusted from 28 to 31 (see Table 2). These newly added categories are beauty care, petroleum and petrochemical, and environmental protection. Additionally, the original “extractive” industry was divided into “coal” and “petroleum and petrochemical”, while “public utilities” was split into “public utilities” and “environmental protection”.

**Figure 1:**
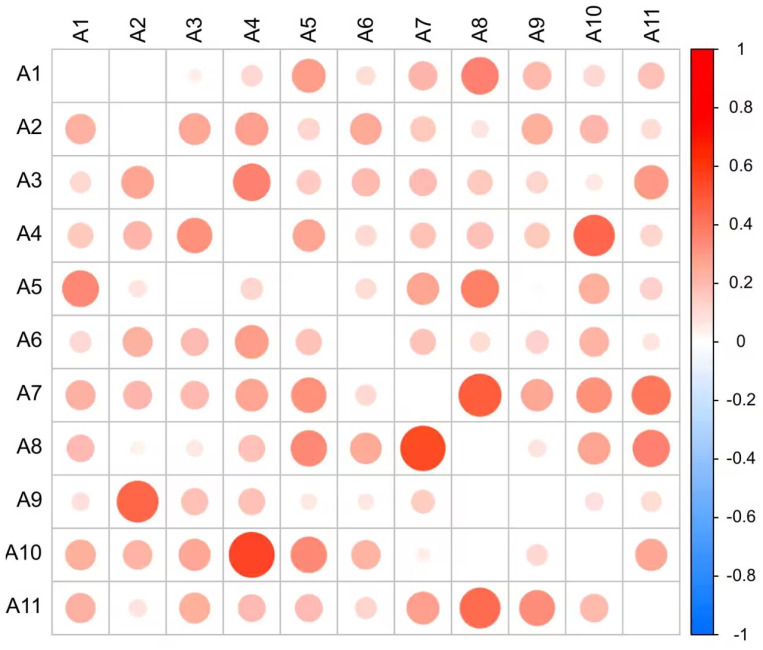
Heat map of PCM causal intensity for the United States industry in 2022 (the size of the circle represents the degree of correlation).

Here, we take industry data from the United States in 2022 as an example to figure out the basic causal linkages (see Figure 1), an interaction matrix for 11 industries is obtained using the PCM technique, and we choose the threshold *T* = 0.25 to pick out the strong linkages among these (for *E* = 2 and τ = 1) [32]. A total of 34 causal combinations were detected, where 18 groups exhibited unidirectional causality; meanwhile, 8 revealed bidirectional causality. The bigger the size of the red circle in the heat map in Figure 1, the higher the degree of the correlation between the two industries, where the horizontal axis is the cause and the vertical axis is the effect.

It is obvious that an extreme mutual causality lies between the industrial and financial sectors in the United States’ industrial system, which is consistent with its status as the world’s industrial and financial power. The communication equipment and finance industries are the two key industries in the United States economic system.

In the Chinese industrial system, the pillar industries have changed according to the Chinese government’s policies and regulations; here, we measure three centrality indicators, as shown in Table 3. The comprehensive industry, with the highest degree centrality (0.5333), and the non-bank financial sector, leading in closeness centrality (0.1063), emerge as central hubs, facilitating extensive and efficient interactions across the network. The chemical industry, with the highest betweenness centrality (0.4931), serves as a crucial intermediary, enhancing the connectivity among diverse sectors. Light manufacturing; food and beverages; and utilities also demonstrate significant bridging roles with high betweenness centrality, while pharmaceuticals and biotechnology, alongside non-bank financials, exhibit strategic positioning in their high closeness centrality. Peripheral industries like textiles and apparel; computers; and electrical equipment show limited influence, highlighting a hierarchical and interconnected network in which strategic sectors maintain economic robustness and resilience.

The most influential industries are distinguished for both countries (see Table 4). The influential industries have varied significantly, indicating a dynamic industrial landscape, in China. Household appliances and light manufacturing appeared multiple times with a strong presence. Traditional sectors, such as pharmaceuticals, nonferrous metals, and food and beverages, have also been significant. Information technology and consumer staples frequently appear, indicating their sustained influence, in the United States. The prominence of information technology is notable in multiple years. Real estate has emerged as influential in recent years, reflecting its growing impact, which underscores the importance of innovation and consumer markets to the United States’ economy.

### 3.2. The Causal Network via PCM

**Figure 2:**
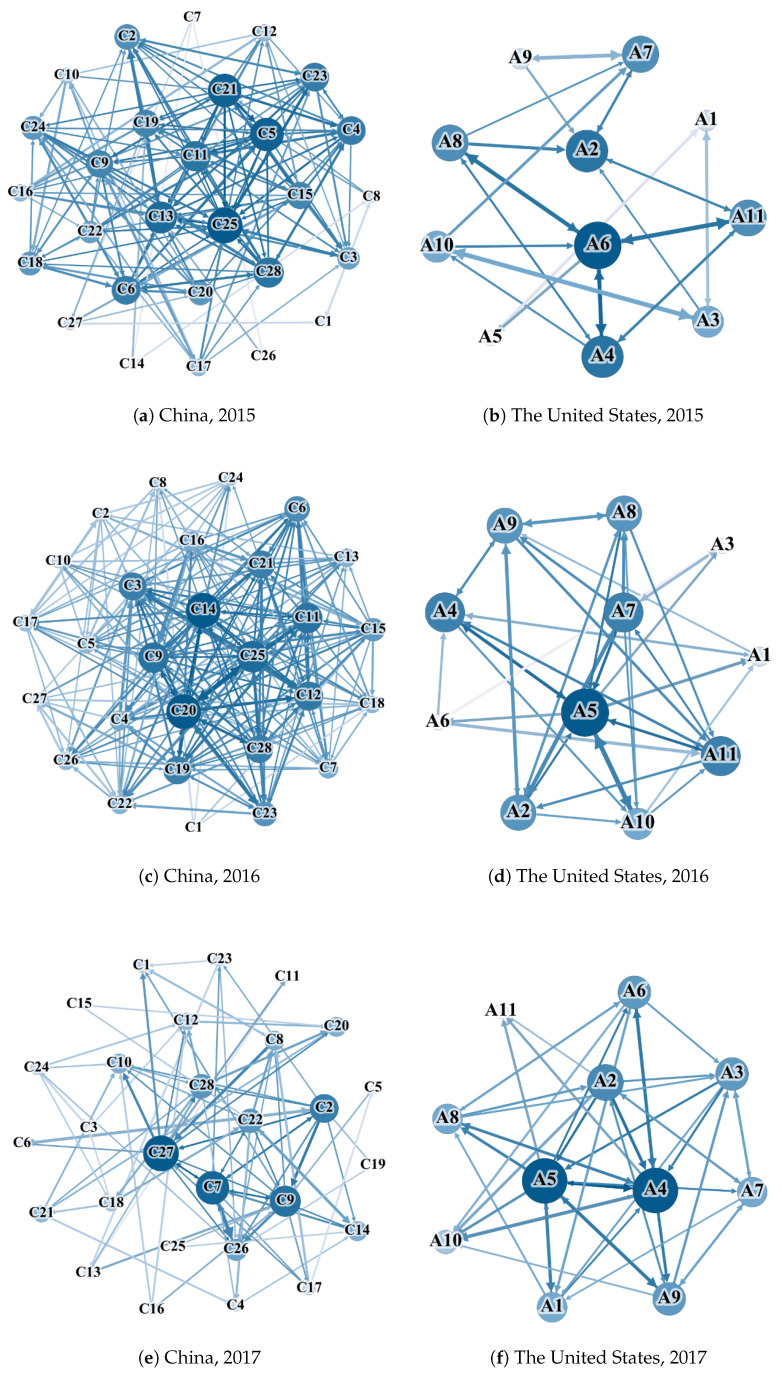

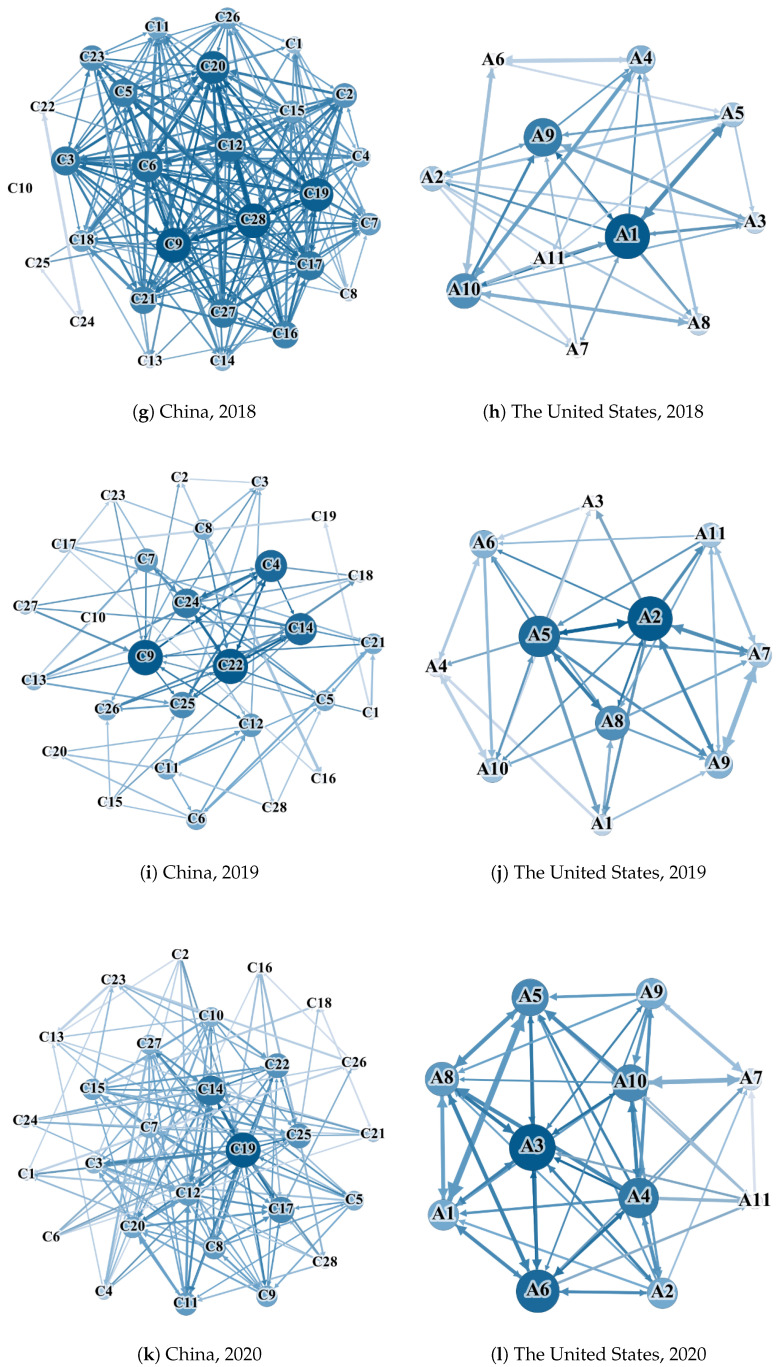

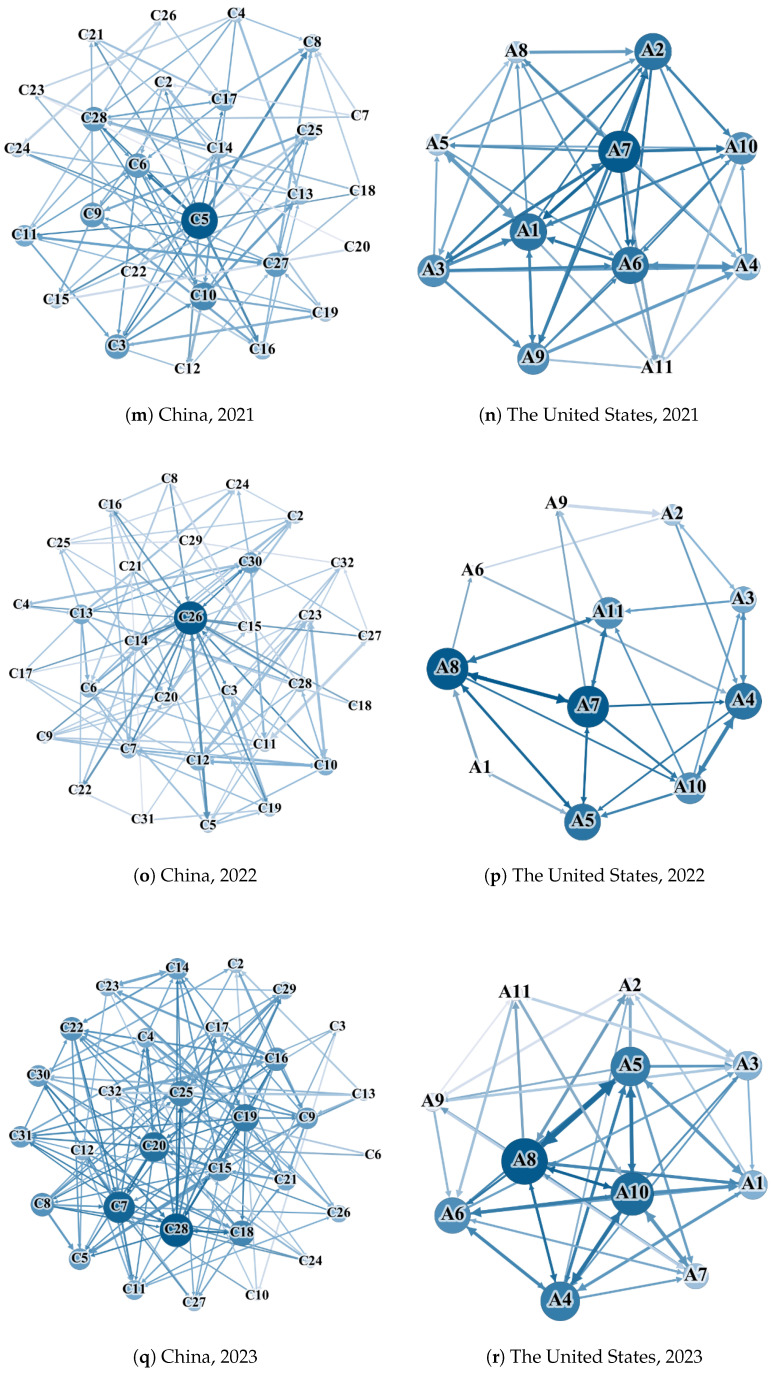
Causal networks of industries for China and the United States (2015–2023).

In this section, we construct the causal network using PCM to detect the linkage structure of industries for both countries. Figure 2 shows the industry interconnection network between China and the United States, with each node representing an industry. The size and color depth of the nodes represent the degree of their criticality in the network. The larger the node and the darker the color, the higher its centrality and influence in the whole network.

In the industry linkage network of different periods, we find that the industry linkage networks for different periods are intricate, and the central industry nodes are constantly changing from 2015 to 2023. Firstly, for China, in 2015 (see Figure 2a), traditional Chinese medicine biology, commercial trade, computers, and the chemical industry were the most influential industries, while household appliances, light industrial manufacturing, and the pharmaceutical and biological industries led in the overall linkage in 2016 (see Figure 2c). In 2017 (see Figure 2e), non-ferrous metals, non-banking finance, and public utilities formed a small community structure in the industry network, affecting the other nodes in the network, which may have been caused by the establishment of the China Non-ferrous Metals International Production Capacity Cooperation Enterprise Alliance (hereinafter referred to as the “alliance”) in early 2017. This alliance was approved by the National Development and Reform Commission and initiated by the China Non-Ferrous Metals Industry Association, under the guidance of the spirit of the 19th National Congress. In 2018 (see Figure 2g), industries blossomed; food and beverages and public utilities were the key industries in 2019 (see Figure 2i). The medical industry became the most influential industry throughout the year of 2020 (see Figure 2k). In 2021 (see Figure 2m), the real estate market declined significantly. As the center of the network, banks were closely connected with other industries in 2022 (see Figure 2o), and their industrial linkage radiated through the whole world. In 2023 (see Figure 2q), the network of industrial linkages in China exhibits significant changes in its structural composition and influence, which reveals that the most prominent nodes within the network are the non-bank financial, steel, and comprehensive sectors. These sectors act as major hubs, exerting considerable influence on the network dynamics and showcasing robust interconnections with other industries. The dominance of these sectors suggests a shift in economic centrality, with their activities radiating outwards and impacting a broad range of industries both domestically and globally. This interconnectedness underscores the pivotal role of these sectors in driving economic activities and shaping the industrial landscape.

The industrial landscape in the United States has undergone notable transformations, with different sectors emerging as pivotal nodes each year. In 2015 (see Figure 2b), materials and consumer staples were central, succeeded by information technology and real estate in 2016 (see Figure 2d). In 2017 (see Figure 2f), information technology and industrials took prominence, while the consumer discretionary and utilities sectors led in 2018 (see Figure 2h). Information technology and consumer staples re-emerged in 2019 (see Figure 2j), followed by health care and materials in 2020 (see Figure 2l). Real estate, consumer discretionary, and materials became key in 2021 (see Figure 2n), with real estate and communication services leading in 2022 (see Figure 2p). Finally, in 2023 (see Figure 2r), communication services, financials, and information technology became the dominant sectors. This dynamic evolution highlights the shifting importance and interconnectedness of various industries over time, reflecting broader economic trends and technological advancements.

### 3.3. Comparation of the Topological Features of the Industrial Networks Between China and the United States

**Figure 3:**
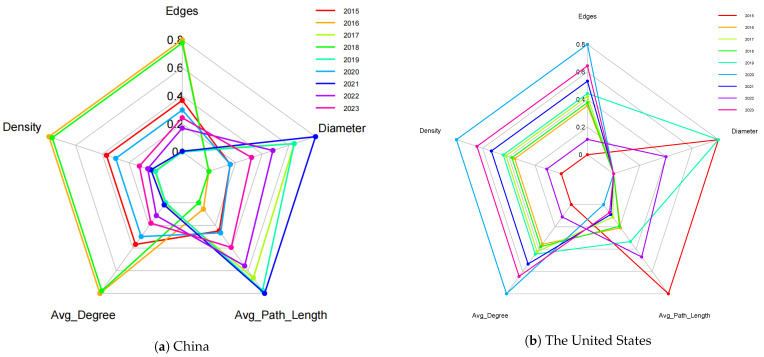
Radar chart for network topology features by year.

The industrial networks of China and the United States exhibit distinct topological characteristics (Figure 3), reflecting the unique structural dynamics, economic strategies, and resilience mechanisms in each country. By examining key metrics such as the edge count, network density, average degree, average path length, and network diameter over time, this study highlights the critical differences and similarities between the two networks.

The edge count serves as a proxy for the overall connectivity within the network. It is evident that the industrial linkage in China (Figure 3a) exhibits a higher number of edges compared to that ub the United States (Figure 3b). For example, in 2016, China’s industrial network recorded 263 edges, significantly surpassing the United States’ 45 edges in the same year. This disparity indicates that China’s network fosters more inter-industry linkages, likely driven by its focus on manufacturing and export-oriented growth. In contrast, the United States network exhibits relatively stable and lower edge counts, peaking at 65 edges in 2020, which reflects its focus on high-value-added sectors with fewer but stronger connections.

Network density measures the proportion of possible connections that are realized within the network. While the United States’ network demonstrates higher densities throughout the period, peaking at 0.591 in 2020, China’s network density fluctuates more significantly, with values ranging from 0.118 in 2019 to 0.348 in 2016. These differences underscore the United States network’s tendency toward compact and efficient connections, while China’s network adapts dynamically to the economic conditions, particularly in response to external shocks such as the Sino–US trade conflict.

The average degree, reflecting the mean number of connections per node, highlights the differences in inter-industry collaboration. China’s average degree varies considerably, reaching a high of 9.393 in 2016 and a low of 3.179 in 2019, indicating episodic surges in connectivity, likely driven by government policies or external shocks. By comparison, the United States network exhibits a more gradual and consistent increase, with the average degree rising from 2.636 in 2015 to 5.273 in 2023. This stability in the United States network points to sustained emphasis on integrated value chains and strategic inter-industry partnerships.

Average path length indicates the efficiency of the information or resource flow within the network. The United States consistently demonstrates shorter path lengths, ranging from 1.418 in 2020 to 2.427 in 2015, signifying a more compact network that facilitates rapid inter-industry communication. In contrast, China exhibits longer path lengths in certain years, such as 2.888 in 2017 and 3.089 in 2019, reflecting a more distributed network structure. However, the shorter path lengths in years like 2016 (1.79) suggest periods of heightened efficiency and tighter interconnectivity.

**Figure 4:**
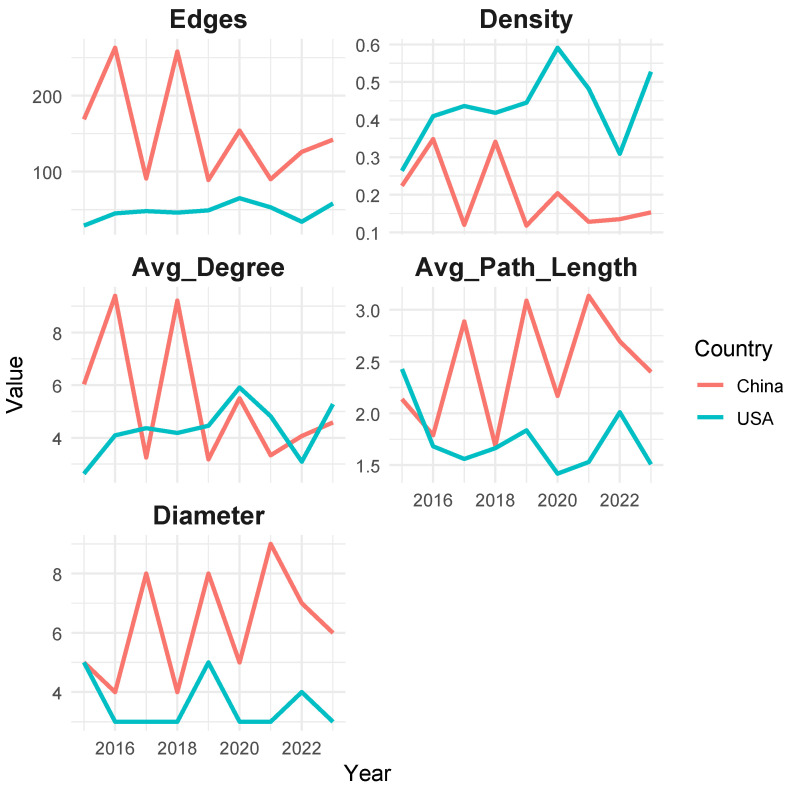
Comparison of network topology features of China and the United States.

Network diameter, the longest distance between any two nodes, offers insights into the network’s overall spread and connectivity. Both networks show variation in their diameters, with China’s ranging from 4 in 2016 to 9 in 2021 and the United States varying between 3 and 5 throughout the period (Figure 4). The larger diameters in China’s network during certain years suggest a broader and more hierarchical structure, while the consistently smaller diameters in the United States network indicate a more compact and centralized configuration.

### 3.4. Network Resilience

**Figure 5:**
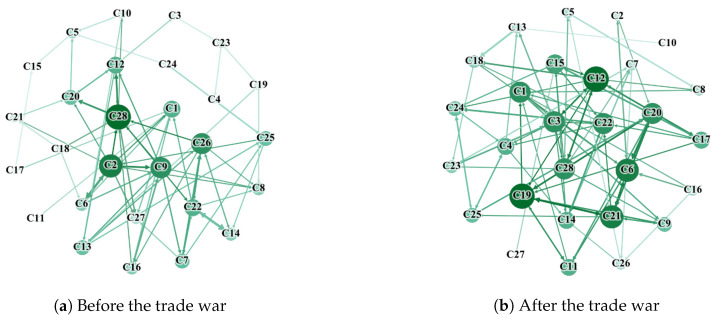
Causal network diagram of PCM of China’s Shenwan industries.

To elucidate the impact of the Sino–US trade war on the causal linkage effects within industry, here, we take China as example and analyze two distinct periods: January 2017 to June 2018 (361 data sets) and July 2018 to December 2019 (368 data sets). We conducted partial cross mapping causal tests for these periods. Figure 5 presents the causal networks from PCM before and after the trade war, while the network topology characteristics are detailed in Table 5.

It is evident that the trade war intensified the connectivity between industry nodes, with the number of connected edges increasing from 69 to 90 (Table 5). This led to a rise in the average number of intermediaries and an increase in the average clustering coefficient from 0.084 to 0.189, which reflected the ‘clustering’ phenomenon among industries; a higher value indicates a stronger connection among industries. Consequently, the overall network density rose from 0.091 to 0.120, which shows the Sino–US trade war enhancing cooperation and connectivity much more than ever before.

We then employed two network disintegration strategies to assess the impact of the Sino–US trade war on the industrial system, using a deliberate attack and a random attack, until the network was fully dismantled. A deliberate attack involves sequentially removing nodes based on their importance, ranked by the number of connections. In contrast, random attacks involve the random removal of nodes to disrupt the network’s connectivity.

**Figure 6:**
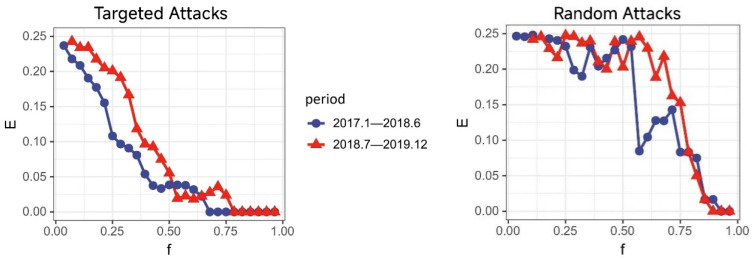
Global network connectivity efficiency.

**Figure 7:**
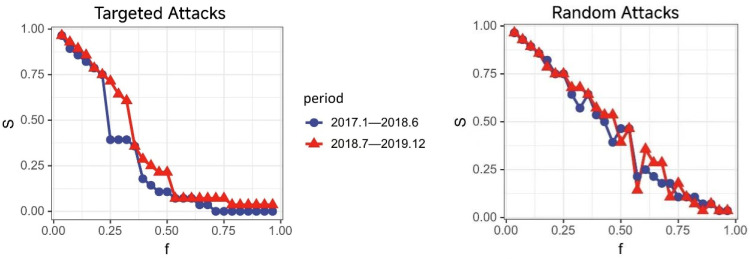
Maximum connectivity subgraph scale efficiency.

Figure 6 and Figure 7 illustrate the changes in the global network connectivity efficiency (E) and the maximum connected subgraph scale (S) over two periods, which demonstrate robust resilience in the post-Sino–US trade war period. This provides insights into how such disruptions could affect network connectivity and stability. The trade conflict acts as an external shock that may alter the importance of certain nodes (industries) or disrupt the causal linkages within the network. By modeling these changes through network failure scenarios, the ability of the industrial network to withstand and recover from disruptions was assessed.

We measure the critical value for network collapse ($f_{0}$) under deliberate attacks as exceeding 0.75 (The threshold $f_{0}$ = 0.75 represents the proportion of nodes removed before network collapse, reflecting network robustness. This value is consistent with studies such as [33], which assessed the error and attack tolerance in complex networks. Similarly, ref. [34] highlights $f_{0}$ as a critical indicator of network resilience in urban systems. While $f_{0}$ = 0.75 involves subjective elements, its adoption in this study provides a standardized measure for comparing network stability.), which shows that the network withstands failure for longer compared to the pre-trade war period. The higher $f_{0}$ in the post-trade war period indicates greater resilience, showing the network can sustain functionality despite node failures. This reflects stronger interconnections and structural robustness within the Chinese industrial network after the trade conflict.

Additionally, the *E* and *S* values in the post-trade war period consistently surpass those from the pre-trade war period, suggesting a more tightly knit network with numerous prominent central nodes. During random attacks, the *E* and *S* value curves post-trade war also generally remain higher, with $f_{0}$ in both periods above 0.82, exhibiting stronger resistance to random disruptions following the Sino–US trade war.

Under both attack modes, the post-Sino–US trade war network demonstrates greater resilience and robustness. This statement about global trade network resilience is based on the observed robustness, as shown in Figure 4. The global implications suggest that such resilience strategies could benefit other nations facing similar trade conflicts.

This enhanced resilience suggests that the trade war fostered closer cooperation within China’s internal industries, thereby strengthening the network’s robustness. The alignment of common interests and relevant policies has fostered a greater propensity for collaboration among Chinese industries, promoting joint efforts in sharing the risks and rewards when facing external challenges. Such close collaboration facilitates resource sharing and information exchange with mutual support, thereby improving the overall resilience of the industrial network to external shocks. Unfortunately, we encountered insufficient nodal points in the industry data series for the United States, which limited the scope of our network resilience analysis.

## 4. Conclusions and Discussion

In this study, we have explored the dependencies and causal relationships within and between the industrial networks of China and the United States, offering a comparative perspective on their structural dynamics and economic resilience. Utilizing the partial cross mapping (PCM) technique alongside complex network theory, we have analyzed real data spanning 2015 to 2023 for 32 industries in China and 11 industries in the United States. Our findings reveal that the Sino–US trade conflict has influenced domestic industrial structures, intensified inter-industry linkages, and reshaped the resilience of industrial networks. In particular, China’s industrial network demonstrates closer connections between nodes, bolstering its stability and robustness in the post-trade war period. These observations align with the broader theoretical understanding that increased connectivity can enhance network resilience while also exposing vulnerabilities to systemic risks [35]. Furthermore, the comparative analysis underscores significant structural differences: the United States exhibits a diversified, service-oriented network dominated by the technology and financial sectors, whereas China’s industrial network emphasizes heavy industries and manufacturing, driven by export-oriented growth. These structural dynamics reflect contrasting economic strategies and their implications for long-term resilience and adaptability.

While this study has illuminated the dynamics of the industrial networks in China and the United States amid the Sino–US trade war, it is crucial to recognize the inherent limitations of the scope and granularity of the data utilized. The analysis relies on industry indices and aggregated data, which, while representative, may not fully capture the intricate and evolving dynamics of industrial networks. Future studies could integrate broader geopolitical and socioeconomic contexts, enhancing the depth of their insights and yielding a more nuanced understanding of the underlying patterns. For instance, the inclusion of global value chain interactions, as highlighted by Baldwin and Lopez-Gonzalez [36], could shed light on how international networks adapt to trade tensions and other systemic shocks.

Moreover, digital transformation continues to reshape industrial linkages, as emphasized by [37,38]. Exploring the impact of technological advancements on industrial networks is a promising direction for future research, as it could reveal new causal patterns and dependencies critical to understanding economic resilience.

The comparative analysis presented in this study underscores how distinct economic structures and policy frameworks shape industrial linkages and stability. The findings demonstrate that China’s industrial network, with its focus on manufacturing and export-driven growth, differs significantly from the United States’ diversified and service-oriented structure. These distinctions provide actionable insights for policymakers, economists, and business leaders in developing strategies to enhance economic resilience and manage risks. For example, understanding the unique characteristics of a country’s industrial network can inform targeted interventions to reinforce economic structures and mitigate potential disruptions.

Nevertheless, it is essential to approach these findings with caution. This study does not claim to fully account for all variables influencing industrial networks during the Sino–US trade war, such as international trade barriers, policy shifts, and external economic shocks. Future research could address these factors by incorporating more granular data, extending the temporal scope, and employing advanced econometric and network models. Such approaches would enable a deeper examination of the resilience and adaptability of industrial networks under varying conditions.

This research also highlights the broader implications of understanding industrial networks. By mapping the causal relationships and identifying key sectors, this study contributes to the development of frameworks applicable to other economies. As noted by [39,40], the complexity of economic networks necessitates sophisticated analytical tools to uncover the structures that drive economic growth and resilience. Policymakers can leverage these insights to design adaptive strategies that foster economic stability and growth, even in the face of global trade tensions and systemic risks.

In conclusion, this study offers a foundation for further exploration of industrial network dynamics, emphasizing the need for interdisciplinary approaches that integrate network theory, policy analyses, and technological insights. By addressing its limitations and building on its findings, future research can contribute to a deeper understanding of global economic systems and inform the creation of resilient and adaptive industrial policies capable of navigating an increasingly interconnected and complex global landscape.

## Figures and Tables

**Table 1:** Industry names and codes in the United States.

Code	Name of Industry	Code	Name of Industry
A1	Consumer Discretionary	A7	Real Estate
A2	Consumer Staples	A8	Communication Services
A3	Health Care	A9	Utilities
A4	Industrials	A10	Financials
A5	Information Technology	A11	Energy
A6	Materials		

**Table 2:** Industry names and codes in China.

Code	Name of Industry	Code	Name of Industry
C1	Mining	C17	Transportation
C2	Media	C18	Agriculture, Forestry, Animal
			Husbandry, and Fishery
C3	Electrical Equipment	C19	Automobiles
C4	Electronics	C20	Light Manufacturing
C5	Real Estate	C21	Commercial Trade
C6	Textiles and Apparel	C22	Food and Beverages
C7	Non-Bank Financials	C23	Communications
C8	Steel	C24	Leisure Services
C9	Utilities	C25	Pharmaceuticals and Biotechnology
C10	National Defense and Military	C26	Banking
C11	Chemical	C27	Nonferrous Metals
C12	Machinery and Equipment	C28	Comprehensive
C13	Computers	C29	Coal
C14	Household Appliances	C30	Environmental Protection
C15	Building Materials	C31	Beauty Care
C16	Construction and Decoration	C32	Petroleum and Petrochemical

**Table 3:** Network centrality indicators of Chinese industries in 2023.

Industry	Betweenness	Closeness	Degree
	Centrality	Centrality	Centrality
Comprehensive	0.4784	0.0784	0.5333
Light Manufacturing	0.4752	0.0792	0.4667
Non-Bank Financials	0.4148	0.1063	0.5000
Food and Beverages	0.4506	0.0649	0.3667
Pharmaceuticals and Biotechnology	0.4083	0.0796	0.4000
Automobiles	0.4083	0.0698	0.4333
Building Materials	0.4148	0.0611	0.3667
Utilities	0.4284	0.0562	0.3333
Steel	0.3843	0.0640	0.3667
Agriculture, Forestry, Animal Husbandry, and Fishery	0.3787	0.0610	0.4000
Household Appliances	0.4667	0.0407	0.3333
Real Estate	0.4752	0.0405	0.3333
Commercial Trade	0.4284	0.0554	0.3000
Chemical	0.4931	0.0202	0.3000
Coal	0.4429	0.0428	0.2667
Environmental Protection	0.3630	0.0595	0.3333
Machinery and Equipment	0.4021	0.0487	0.2667
Media	0.3308	0.0734	0.2333
Beauty Care	0.3787	0.0265	0.3333
Banking	0.4148	0.0249	0.2667
Communications	0.3787	0.0479	0.2667
Transportation	0.4284	0.0144	0.2667
Construction and Decoration	0.3149	0.0381	0.3667
Petroleum and Petrochemical	0.3226	0.0562	0.2333
Nonferrous Metals	0.4021	0.0161	0.2333
National Defense and Military	0.3787	0.0474	0.1333
Electronics	0.3630	0.0147	0.3000
Leisure Services	0.3948	0.0000	0.1667
Electrical Equipment	0.3394	0.0103	0.1333
Textiles and Apparel	0.3641	0.0000	0.0667
Computers	0.2465	0.0031	0.1667

**Table 4:** Most influential industries for both countries in previous years.

Year	Two of the Most Influential Industries	Two of the Most Influential Industries
	in China	in the United States
2015	Pharmaceuticals and Biotechnology,	Materials, Consumer Staples
	Commercial Trade	
2016	Household Appliances, Light Manufacturing	Information Technology, Industrials
2017	Nonferrous Metals, Non-Bank Financials	Information Technology, Industrials
2018	Comprehensive, Utilities	Consumer Discretionary, Utilities
2019	Food and Beverages, Utilities	Consumer Staples, Information Technology
2020	Automobiles, Household Appliances	Health Care, Materials
2021	Real Estate, National Defense and Military	Real Estate, Consumer Discretionary
2022	Banking, Computers	Real Estate, Communication Services
2023	Comprehensive, Light Manufacturing	Communication Services, Financials

**Table 5:** Network topology characteristics before and after the trade war.

	Network	Network	Average	Average
	Edges	Density	Betweenness	Clustering Coefficient
Before the start of the US–China trade war	69	0.091	0.063	0.084
After the start of the US–China trade war	90	0.120	0.076	0.189

## Data Availability

The data used to support the findings of this study are available from the corresponding author upon request.
